# Ranking the environmental factors of indoor air quality of metropolitan independent coffee shops by Random Forests model

**DOI:** 10.1038/s41598-022-20421-2

**Published:** 2022-09-26

**Authors:** Yu-Wen Lin, Chin-Sheng Tang, Hsi-Chen Liu, Tzu-Ying Lee, Hsiao-Yun Huang, Tzu-An Hsu, Li-Te Chang

**Affiliations:** 1 Department of Public Health, Fu Jen Catholic University, New Taipei City, Taiwan; 2 Department of Labor and Human Resources, Chinese Culture University, Taipei City, Taiwan; 3 Department of Statistics and Information Science, Fu Jen Catholic University, New Taipei City, Taiwan; 4 Department of Environmental Engineering and Science, Feng Chia University, Taichung City, Taiwan

**Keywords:** Environmental sciences, Occupational health

## Abstract

Independent coffee shops are the alternative workplaces for people working remotely from traditional offices but are not concerned about their indoor air quality (IAQ). This study aimed to rank the environmental factors in affecting the IAQ by Random Forests (RFs) models. The indoor environments and human activities of participated independent coffee shops were observed and recorded for 3 consecutive days including weekdays and weekend during the business hours. The multi-sized particulate matter (PM), particle-bound polycyclic aromatic hydrocarbons (p-PAHs), total volatile organic compounds (TVOCs), CO, CO_2_, temperature and relative humidity were monitored. RFs models ranked the environmental factors. More than 20% of the 15-min average concentrations of PM_10_, PM_2.5_, and CO_2_ exceeded the World Health Organization guidelines. Occupant density affected TVOCs, p-PAHs and CO_2_ concentrations directly. Tobacco smoking dominated PM_10_, PM_2.5_, TVOCs and p-PAHs concentrations mostly. CO concentration was affected by roasting bean first and tobacco smoking secondly. The non-linear relationships between temperature and these pollutants illustrated the relative low concentrations happened at temperature between 22 and 24 °C. Tobacco smoking, roasting beans and occupant density are the observable activities to alert the IAQ change. Decreasing CO_2_ and optimizing the room temperature could also be the surrogate parameters to assure the IAQ.

## Introduction

People spend 80–90% of their time in indoor environments, such as homes or workplaces. Therefore, health effects caused by indoor air quality (IAQ) should be addressed. Particulate matter, with aerodynamic diameters ≤ 2.5 μm (PM_2.5_) and 10 μm (PM_10_), is the major concerned pollutant in the IAQ. Study showed that most of the indoor PM_2.5_ concentrations were higher than the concentrations of outdoor^1^. In addition, total volatile organic compounds (TVOCs), particle-bound polycyclic aromatic hydrocarbons (p-PAHs) and pollutants from burning solid fuels, such as carbon dioxide (CO_2_) and carbon monoxide (CO), are also major indoor air pollutants (IAPs)^2,3^.

The p-PAHs and VOCs were detected in coffee shops^3–5^. VOCs plays the role in the perception of order and favor of food. Various VOCs were detected in the headspace of brewing espresso coffee^6^. The Global Workplace Analytics estimates around 4.3 million people work remote at least half the time and as a result the traditional office setting is being replaced by alternative workspaces—the readily-available independent coffee shop is one of choices^7^. More than 70% of independent café consumers surveyed purchase coffee to drink in-store. It was estimated that independent stores served more than 10.5 million cups of coffee each week in UK^8^. So, it is important to understand the IAQ in independent coffee shops as they are served as “workplaces” and the consumers’ preferring indoor environment. Besides characteristics of chain coffee shops, independent coffee shops usually roast their own beans on sites. Hence, the levels of CO^9^ and VOCs^6,10^ in independent coffee shops shall be addressed.

Some indoor environmental factors affected IAQ. For example, environmental tobacco smoke (ETS) would change the IAQ mentioned in several researches^1,11–13^ as well as human activities^1,14–16^ and occupant density^17,18^. PM and TVOCs concentration was associated with indoor air flow, temperature, and relative humidity (RH)^19–21^; in addition, it was associated with level of p-PAHs^22^. As a better surrogate of ventilation efficiency and IAQ indicator, CO_2 _could be used to represent other pollutants in indoor air other than PM could^23–28^. Therefore, those indoor environmental factors mentioned above might be the indicators to remind staffs of coffee shops to notice IAQ. However, there were few studies assessed which environmental factors would be the useful and simple indicators for IAQ.

Random Forests (RFs) is a machine learning or statistical learning model^29^. Some studies investigated factors associated with selected IAPs by RFs analysis and proofed that RFs models had better abilities in prediction than multiple linear regression or other methods^30–32^. RFs is a data driven method to explore relationships when the independent and the dependent variables have non-linear relationships^33,34^, and the definition of the importance of variables is not based on the estimation of coefficients, which avoids important variables be ignored due to estimation problems^35–37^. Furthermore, RFs has relatively low requirements for the completeness of the data^33,34^. RFs seldom used in investigating the importance of factors associated with IAQ. This study continuously monitored the IAPs and indoor environment during the business hours of the investigated independent coffee shops and aimed to demonstrate how the multiple environmental factors affect the IAQ by RFs models. The RFs models ranked the importance of the factors and suggested the proactive indicators for the IAQ levels in these novel workplaces.

## Methods

### Recruiting participated coffee shops

We recruited independent coffee shops in the metropolitan area of Taipei, Taiwan and four shops (labeled as A, B, C, and D) participated in this study. The investigations were proceeded from November 2019 to March 2020. At the beginning of each on-site monitoring day, our team members obtained the shops’ information including the business hours, the floor plan, type of building, indoor space volume, building materials, smoking area design, ventilation equipment, and window opening situation. The detailed characteristics was listed in Supplement A.

### Indoor environments, air pollutants, and meteorological monitoring

Indoor environments of participated shops were collected by activity log and direct-reading instruments. Our team members filled in the activity log on the monitoring day which included the nature of the indoor activity, ventilation status, and the numbers of people with a 15-min interval. The indoor activities included cooking, roasting beans, cleaning, tobacco smoking, and other behaviors that might change the air quality. The members rechecked the information to assure the correctness after completion. The monitoring was proceeded continuously during the business hours for 3 consecutive days including weekdays and weekends in each coffee shop. A fixed sampling point was arranged to meet the 3-day measurement requirement without interrupting the normal business. The sampling point was not changed during sampling times to eliminate the interferences of sampling point, such as air flow, the distance from the kitchen area to the sampling point, and the distance from the entrance door to the sampling point.

**Figure 1:**
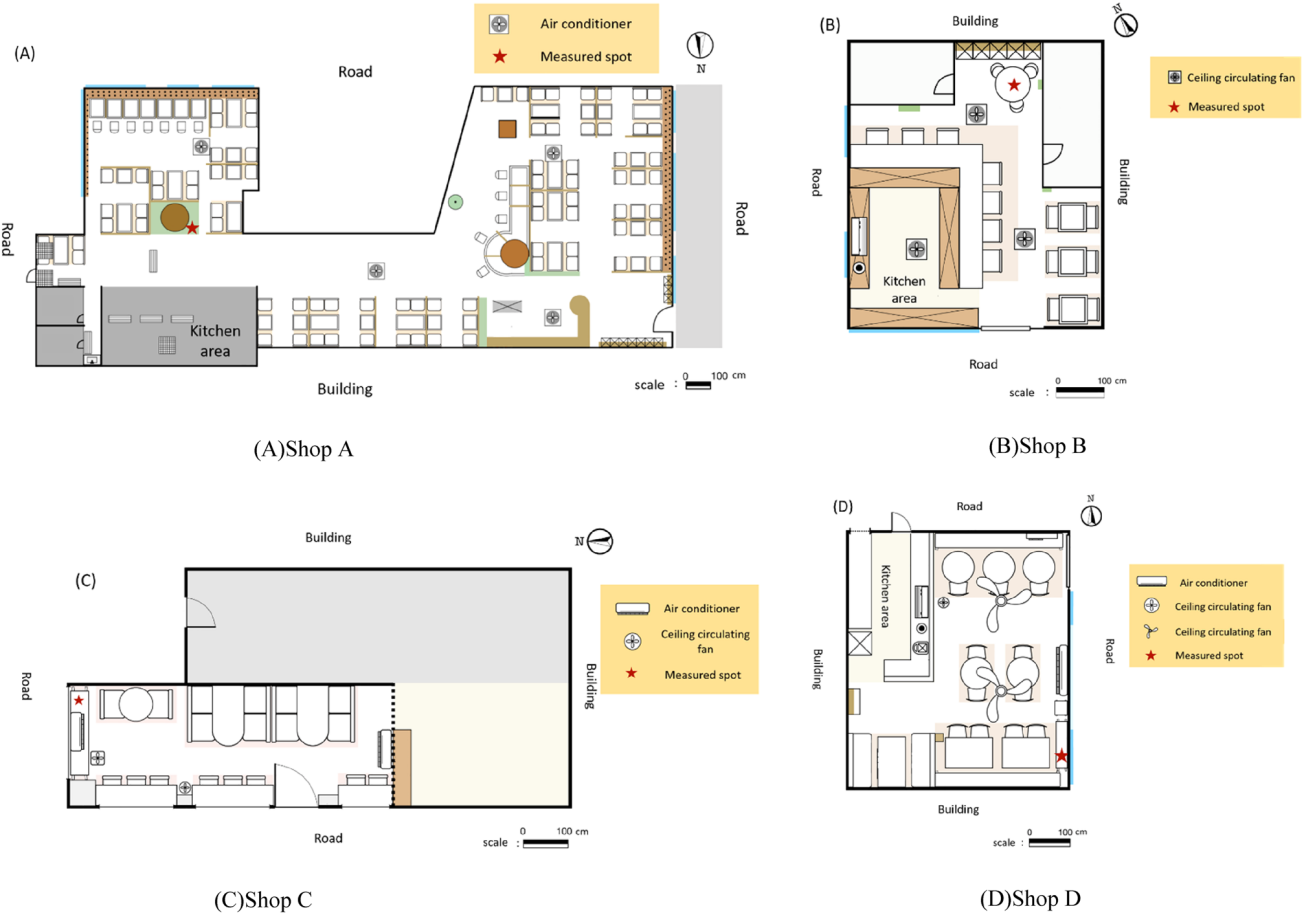
Floor plans and sampling points of four coffee shops.

A portable IAQ monitor (Smart Indoor Air Quality Sensing Controller Model GiA-K007, NewGreen Tech Co., Taiwan) was employed to monitor temperature and RH continuously. This IAQ monitor can also measure CO by a built-in electrochemical CO sensor with a detection range of 0–500 ppm and CO_2_ by a NDIR CO_2_ sensor with a detection range of 0–10,000 ppm. The portable aerosol analyzer (Model 1.108, Grimm Aerosol Technik GmbH & Co. KG, Ainring, Germany) was used to measure the PMs at a flow rate of 1.2 L/min. The mass concentrations of PM_10_ and PM_2.5_ were selected. Mass concentrations of PM_2.5–10_ (coarse PM) were obtained by subtracting the PM_2.5_ fraction from the concurrent PM_10_ levels. A photoelectric aerosol sensor (PAS2000CE, EcoChem Analytics, League City, TX, USA) was used to measure indoor p-PAHs level with the detection range of 0–4000 ng/m^3^. The PAS2000CE measures the PAHs with more than three rings adsorbed onto the approximately 1-μm carbonaceous particles^5^. In addition, we used a ppbRAE 3000 photoionization detector (PID) (model ppbRAE 3000; RAE systems, Inc., USA) with a 10.6 eV lamp with an extended range of 1–10,000 ppm to quantify TVOCs. All the monitoring instruments were set to output one value every minute. In addition to the routine calibration and maintenance of the instruments used in this study, the research staff also performed essential calibration for the instrument readings and pump flows before and after each field survey. The sampling spots (i.e., the location of the instruments) in each shop were shown in Fig. 1.

### Statistical analysis

To ensure the quality of data processing, Microsoft Excel (2019) was used for data management and descriptive analysis. Zero, negative, missing, and unreasonably high and low values were excluded, as were continuous values in a range more than ten folds. The data (pollutants’ concentrations and meteorological data) were synchronized with the people counts. The concentrations were expressed as a 15-min average and the occupant density was the number of indoor people counts dividing by the floor area.

In this study, the R's package (R, 3.5.1) ‘randomForest’^38^ was used to build RFs models to examine the importance of indoor environmental factors associated with specific IAPs. The environmental factors included coffee shop, weekday, occupant density, indoor activities, ventilation status, locating on the main traffic street, and meteorological parameters (temperature and RH). The meteorological parameters were defined as the independent variables in the RFs models, as the IAPs, such as PMs and TVOCs, were affected by these parameters^19–21,23,39–42^. As a surrogate of air change rate and ventilation efficiency^43–45^, CO_2_ concentration was also served as a potential determinant of other IAPs^23–28^. The 15-min average concentrations of IAPs were the dependent variables.

RFs model is to repeatedly generate multiple bootstrapping sample sets that are different from each other by the bootstrapping method from the training samples. A decision tree model is established according to each bootstrapping sample set, which about two-thirds of the training samples. Then, about one-third of the samples are still not selected, being referred to as out-of-bag (OOB) samples^33^. It can be used as a testing sample to measure the generalization performance of the model by estimating the OOB error. According to the prior analysis, the number of decision trees of each RFs model is set to 500 to obtain the convergent OOB error. The “rfcv” code of RFs was applied to rank and plot importance of the variables^46^. RFs measured the importance of variables within its model building mechanism. During the construction of the RFs model, the impurity reduced by the addition of each node variable in each decision tree was calculated, and finally, the average of all reduced impurity of each variable was reported. The mean decrease in node impurity of each variable was used as a measure of the importance of the variable^33^. The mean decrease in node impurity is denoted by “IncNodePurity” as shown in all result figures of the variable importance rank. Then, the partial dependence plots of the independent variables were plotted by “partial Plot” code of RFs. The partial plot illustrated the relationship between a specific independent variable and all dependent variables by controlling other dependent variables^29,46^. The partial dependence plot indicated the percentiles of the independent variable (x-axis) as “rug” for continuous variable. We observed the relationship between the 10th and 90th percentiles of the independent variable and the correspondent dependent variable.

## Results

### Concentrations of indoor air pollutants

Table 1 showed the concentrations of IAPs and indoor meteorological parameters (temperature and RH). Although the average concentrations of IAPs were low, the mean of PM_2.5_ concentrations of shop C exceeded the 24-h average PM_2.5_ of Taiwan Environmental Protection Administration (EPA)^47^ IAQ standards 35 μg/m^3^ and the World Health Organization (WHO)^48^ guidelines 15 μg/m^3^. The 15-min averages of IAPs were compared to the Taiwan IAQ standards and WHO guidelines. The PM_2.5_ 15-min averages of coffee shop B, C and D had 0.0%, 25.3% and 1.2% exceeded the Taiwan IAQ standard 35 μg/m^3^ and 13.6%, 29.5% and 12.1% exceeded the WHO IAQ guidelines 15 μg/m^3^ respectively. The portions of 5%, 2.3% and 21.1% of the PM_10_ 15-min averages of coffee shop A, B and C exceeded 75 μg/m^3^ (Taiwan IAQ standard of 24-h average PM_10_) accordingly. The PM_10_ 15-min averages of all investigated coffee shops exceeded the WHO guidelines of 24-h average PM_10_ 45 μg/m^3^ with the portions of A 5.0%, B 4.5%, C 24.2%, and D 1.2%. The CO_2_ 15-min averages of coffee shop A, C, and D showed 5%, 21.1%, and 36.1% exceeded 1000 ppm (Taiwan IAQ standard of 8-h average CO_2_). For 15-min averages of TVOCs, coffee C and D shop exceeded 0.56 ppm (Taiwan IAQ standard of 1-h average TVOCs) in the portions of 20% and 1.2% respectively. For comfort parameters, all participated coffee shops controlled the temperature below 26 °C, but the 15-min averages of RH for all shops exceeded 70% with the portions of 100.0%, 34.1%, 23.2% and 55.4% for shop A, B, C, and D accordingly. The RH was set at 70% during occupancy by most Asian countries IAQ standards and guidelines^49^.

**Table 1:** Descriptive statistics of the concentrations^a^ of indoor air pollutants and meteorological data^a^ in the studied coffee shops.

	Shop A (N = 20)		Shop B (N = 44)		Shop C (N = 95)		Shop D (N = 83)	
	Range	Mean ± SD	Range	Mean ± SD	Range	Mean ± SD	Range	Mean ± SD
PM_10_ (μg/m^3^)	2.3–140.9	10.7 ± 30.6	6.6–80.1	23.2 ± 14.1	7.5–161.3	44.2 ± 44.8	4.0–57.2	13.2 ± 8.0
PM_2.5–10_ (μg/m^3^)	0.3–138.3	7.6 ± 30.7	3.9–72.0	13.7 ± 12.3	2.0–18.1	6.5 ± 3.2	1.6–13.0	5.1 ± 2.1
PM_2.5_ (μg/m^3^)	1.7–5.4	3.1 ± 1.0	2.7–17.9	9.5 ± 4.9	3.9–152.4	35.8 ± 41.9	1.9–48.7	8.1 ± 6.8
Total VOCs (ppm)	NA	NA	0.1–0.3	0.1 ± 0.0	0.0–1.5	0.3 ± 0.4	0.1–0.8	0.2 ± 0.1
p-PAHs (ng/m^3^)	NA	NA	NA	NA	2.6–193.7	20.5 ± 29.3	6.6–24.5	12.4 ± 3.7
CO (ppm)	ND	ND	ND-4.9	0.9 ± 1.4	0.0–4.0	0.5 ± 1.0	ND	ND
CO_2_ (ppm)	679.6–1626.8	785.8 ± 201.5	475.0–838.8	590.1 ± 93.4	442.7–1774.9	771.6 ± 309.2	543.3–1607.4	895.4 ± 245.2
Temperature (°C)	21.8–24.6	23.6 ± 0.8	21.5–24.1	23.3 ± 0.6	19.9–25.4	22.7 ± 1.3	20.0–25.8	22.5 ± 1.7
RH (%)	70.3–80.8	75.7 ± 3.3	53.8–80.4	67.5 ± 8.2	53.0–73.6	65.6 ± 5.7	64.5–81.2	71.7 ± 4.6

PM_10_, particulate matter with an aerodynamic diameter less than 10 μm; PM_2.5–10_, particulate matter with an aerodynamic diameter between 2.5 and 10 μm; PM_2.5_, particulate matter with an aerodynamic diameter less than 2.5 μm; Total VOCs, total volatile organic compounds; p-PAHs, particulate polycyclic aromatic hydrocarbons; CO, carbon monoxide (ppm); CO_2_, carbon dioxide (ppm); RH, relative humidity (%); NA, not available due to instrumental malfunction; ND, not detected.

^a^Concentrations were 15-min averages.

### Characteristics of environmental factors

Characteristics of indoor environmental factors of four coffee shops were listed in Table 2. The indoor activities were counted every 15 min during the on-site surveillance period and summarized as the percent of the total activity counts. The most common indoor activity is cooking with a frequency of 30.0% for Shop A and 21.7% for Shop D. Roasting beans and cleaning are the second frequent indoor activities in these cafes. The major activity was roasting beans for shop B (25.0%). In shop C, 31.6% of the total indoor activities was indoor tobacco smoking. This is the only café allowed indoor tobacco smoking. Indeed, it is prohibited in Taiwan.

**Table 2:** Summary of the indoor environmental characteristics in the investigated coffee shops.

	Shop A (N = 20)		Shop B (N = 44)		Shop C (N = 95)		Shop D (N = 83)	
	n	%	n	%	n	%	n	%
**Indoor activities**^**a**^								
Cooking	6	30.0%	1	2.3%	10	10.5%	18	21.7%
Roasting beans	0	0.0%	11	25.0%	2	2.1%	9	10.8%
Cleaning	0	0.0%	3	6.8%	0	0.0%	7	8.4%
Smoking	0	0.0%	0	0.0%	30	31.6%	0	0.0%
Others	0	0.0%	3	6.8%	0	0.0%	1	1.2%
**Ventilation status**^**a**^								
AC on/Window or door open	0	0.0%	44	100.0%	0	0.0%	0	0.0%
AC on/window or door closed	20	100.0%	0	0.0%	86	90.5%	83	100.0%
AC off/window or door closed	0	0.0%	0	0.0%	9	9.5%	0	0.0%
Locating on the main traffic street	Yes		No		No		No	
Occupant density (person/100 m^2^)	8–13^b^	11 ± 1^c^	7–33^b^	17 ± 6^c^	3–20^b^	10 ± 3^c^	9–66^b^	28 ± 3^c^

AC, air conditioner.

^a^Recorded by 15-min interval. ^b^Range. ^c^Mean ± SD.

The mean occupant density of shop D was 28 occupants/100 m^2^ (range: 9–66 occupants/100 m^2^), higher than the American Society of Heating, Refrigerating, and Air-Conditioning Engineers (ASHRAE) recommendation 20 occupants/100 m^2^ for coffee stations^50^. During the monitoring periods, 15.9% and 61.4% of the 15-min interval exceeded the recommended value (20 occupants/100 m^2^) for coffee shop B and D respectively.

### Ranking the environmental factors by RFs models

**Figure 2:**
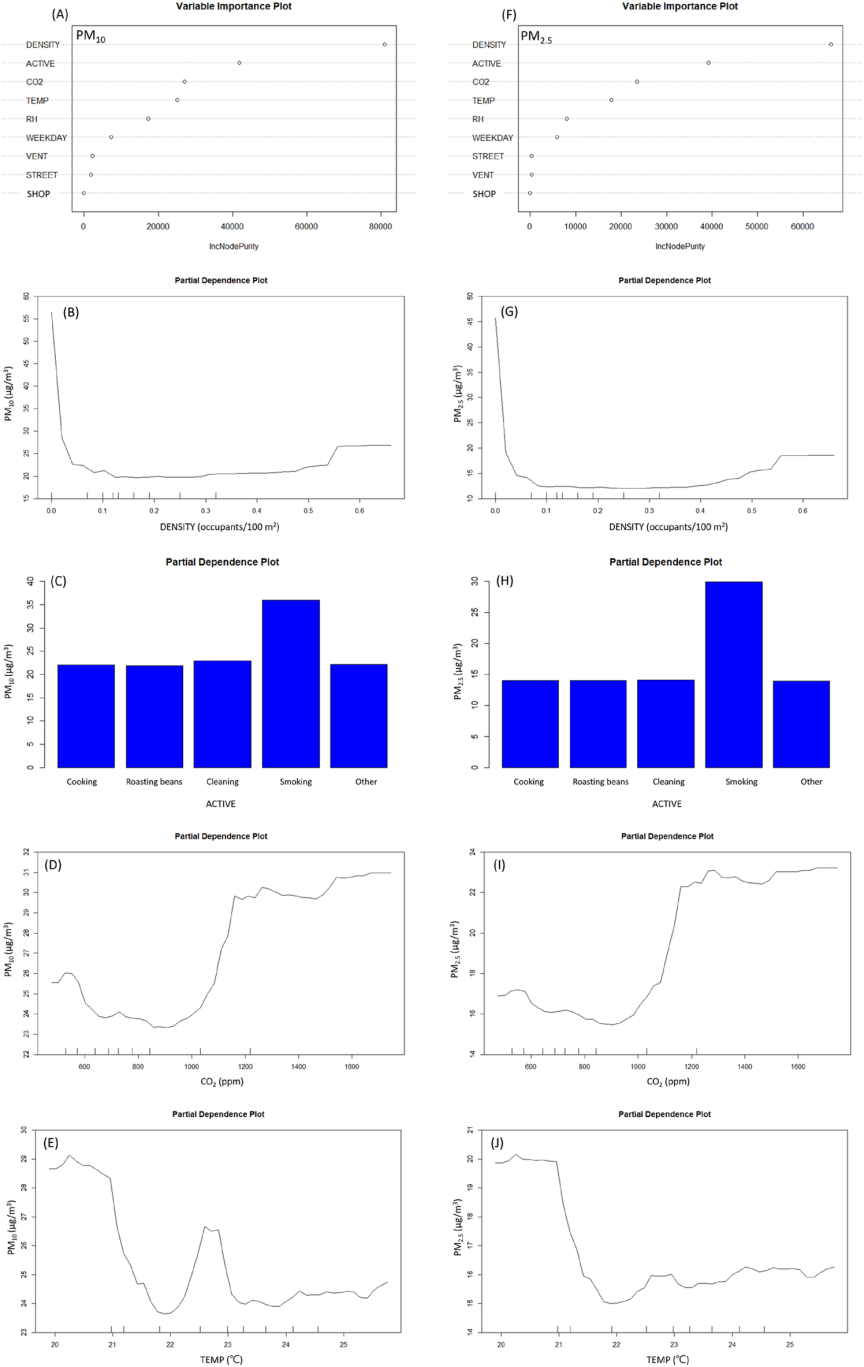
Variable importance rank and partial dependence plot of PM_10_ and PM_2.5_ from Random Forests models. (PM_10_: particulate matter with an aerodynamic diameter ≤ 10 μm (μg/m^3^); PM_2.5_: particulate matter with an aerodynamic diameter ≤ 2.5 μm (μg/m^3^) ACTIVE: Indoor activities; CO_2_: carbon dioxide (ppm); DENSITY: Occupant density (person/m^2^); RH: relative humidity (%); SHOP: coffee shop A, B, C, D; STREET: locating on the main traffic street; TEMP: temperature(°C); VENT: ventilation status).

The occupant density, CO_2_, temperature, indoor activities, and RH were identified as the top 5 indicators by the variable importance plots resulting from the RFs models of each IAP. For PM_10_ and PM_2.5_, occupant density and human activities were the top two indicators (Fig. 2A,F)_._ Excluding the outliers of the occupant density, the concentrations of PM_10_ and PM_2.5_ slightly increased as occupant density increased that was found from the partial dependence plot in Fig. 2B,G. The highest partial average level of PM_10_ and PM_2.5_ occurred during the indoor activity “tobacco smoking” and the difference from other activities were 14 μg/m^3^ and 16 μg/m^3^, respectively (Fig. 2C,H). The CO_2_ concentration was the third important indicator for indoor concentration of PM_10_ and PM_2.5_. When the concentration of CO_2_ increased from 900 ppm to about 1300 ppm, the concentrations of PM_10_ and PM_2.5_ were proportional to the concentration of CO_2_, and the increase concentration of PM_10_ and PM_2.5_ were 6.5 μg/m^3^ and 7.5 μg/m^3^, respectively (Fig. 2D,I). Temperature was the fourth important indicator for PM_10_ and PM_2.5_. The correlations between PM (PM_10_ and PM_2.5_) and temperature are nonlinear. The lowest concentrations happened at 22 °C. Then, the PM concentrations remained stable at 24 μg/m^3^ for PM_10_ and 18 μg/m^3^ for PM_2.5_ as the temperature maintaining at 23–26 °C. T (Fig. 2E,J). The R^2^ of RFs model of PM_10_ and PM_2.5_ were 0.71 and 0.80, respectively.

**Figure 3:**
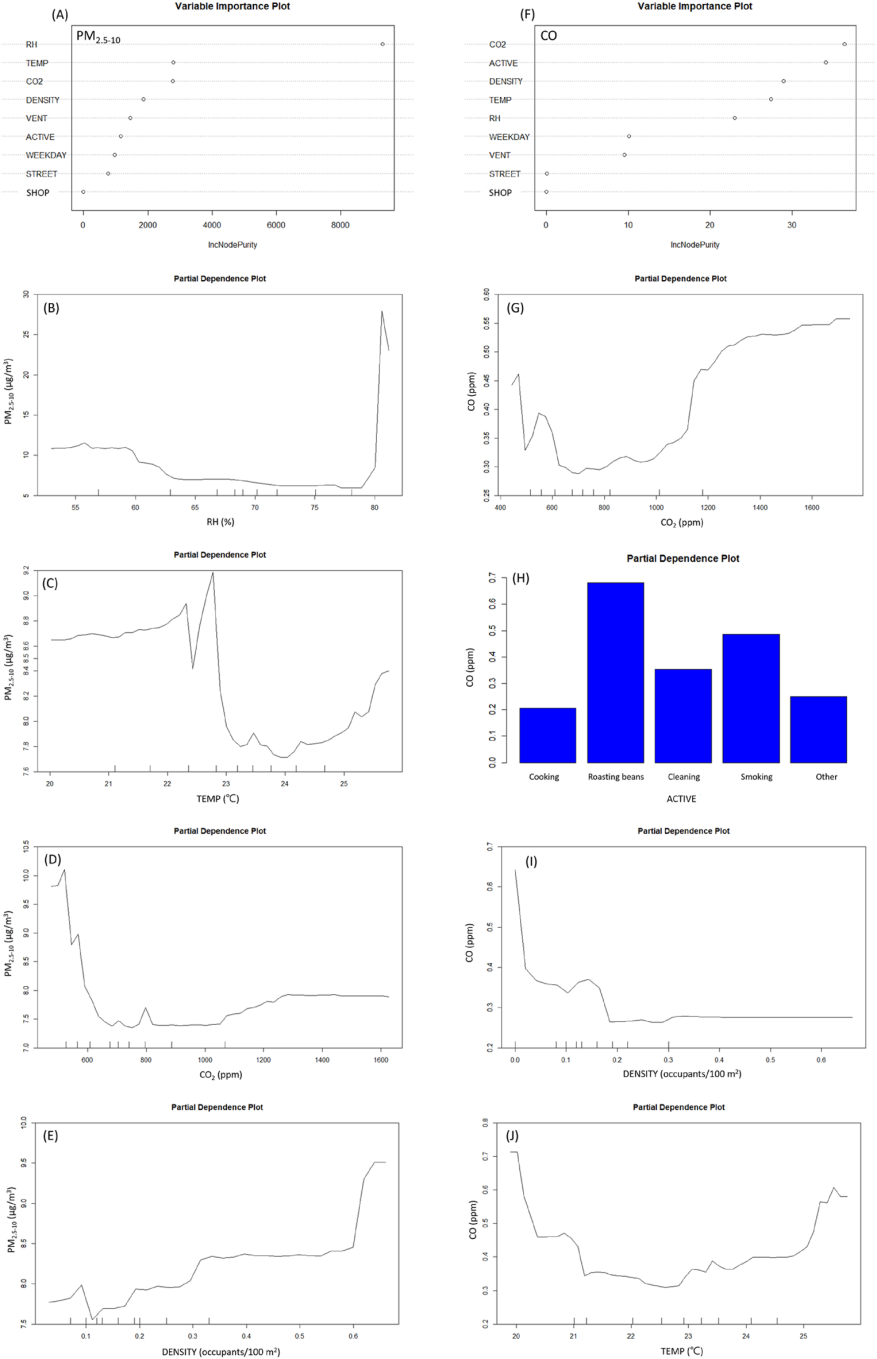
Variable importance rank and partial dependence plot of PM_2.5–10_ and carbon monoxide (CO) from Random Forests models. (PM_2.5–10_, particulate matter with an aerodynamic diameter between 2.5 and < 10 μm (μg/m^3^); ACTIVE, Indoor activities; CO_2_, carbon dioxide(ppm); DENSITY, Occupant density (person/m^2^); RH, relative humidity (%); SHOP, coffee shop A, B, C, D; STREET, locating on the main traffic street; TEMP, temperature(°C); VENT, ventilation status).

The R^2^ of RFs model for PM_2.5–10_ was 0.21 and the top four important indicators were RH, temperature, CO_2_ and occupant density for PM_2.5–10_ (Fig. 3A). Excluding the outlying RH (> 80%), the concentrations of PM_2.5–10_ slightly decreased as RH increased from around 58% to 78% (Fig. 3B). The relationship between temperature and PM_2.5–10_ was shown in “W” shape. The bottom was at about 23.2 °C and 24 °C. When the temperature was lower than 23.2 °C, the relationship between temperature and PM_2.5–10_ was complicated. On the other hand, when the temperature was higher than 24 °C, the concentrations of PM_2.5–10_ was positively proportional to temperature (Fig. 3C). When the concentration of CO_2_ was higher than about 1050 ppm, the concentration of PM_2.5–10_ was proportional to the CO_2_ concentration (Fig. 3D). Excluding the outlying occupant density, the concentrations of PM_2.5–10_ slightly increased as occupant density increased from about 11 to 33 person/100 m^2^ (Fig. 3E).

The top four indicators shown in RFs model for CO were CO_2_, indoor activity, occupant density and temperature (Fig. 3F) with the R^2^ of 0.46. When indoor concentration of CO_2_ was around 680 to 1450 ppm, the concentration of CO was proportional to the concentration of CO_2_ (Fig. 3G). The highest partial average level of CO was occurred with activity “roasting beans” at about 0.7 ppm, and the second highest partial average level of CO was occurred with indoor activity “tobacco smoking” at about 0.5 ppm (Fig. 3H). The CO concentration had a negative relationship with occupant density when the density was lower than 0.3 person/m^2^. When density was higher than 0.3 person/m^2^, the concentration of CO was not affected by the density (Fig. 3I). The relationship between the concentration of CO and temperature was shown in “U” shape. Basically, CO was relatively low when temperature was between 21.2 to 24.5 °C (Fig. 3J).

**Figure 4:**
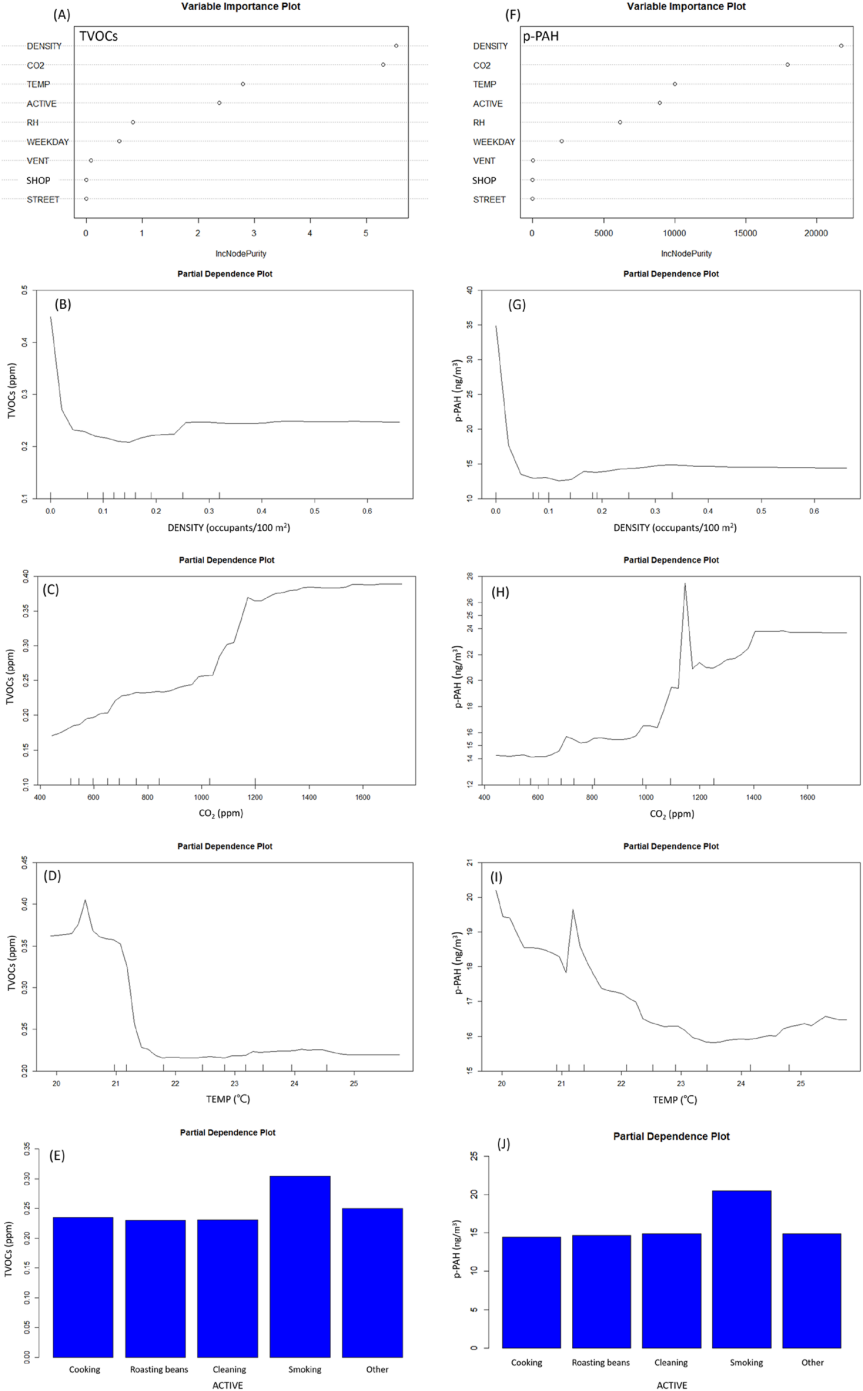
Variable importance rank and partial dependence plot of total volatile organic compounds (TVOCs) and particle-bound polycyclic aromatic hydrocarbons (p-PAHs) from Random Forests models. (ACTIVE, Indoor activities; CO_2_, carbon dioxide (ppm); DENSITY, Occupant density (person/m^2^); RH, relative humidity (%); SHOP, coffee shop A, B, C, D; STREET, locating on the main traffic street; TEMP, temperature(°C); VENT, ventilation status).

Occupant density, CO_2_, temperature, and indoor activities were the top four important indicators for TVOCs and p-PAHs (Fig. 4A,F), and the R^2^ of RFs model were 0.77 and 0.55, respectively. The concentrations of TVOCs and p-PAHs were positively proportional to occupant density between 0.15 and 0.33 person/m^2^ (Fig. 4B,G). When the concentration of CO_2_ was around 450 to 1200 ppm, the concentration of TVOCs was proportional to the concentration of CO_2_ and the increase of TVOCs was about 0.2 ppm (Fig. 4C). When the concentration of CO_2_ was around 500 to 1150 ppm, the concentration of p-PAHs was proportional to the concentration of CO_2_ and the increase of p-PAHs was about 13.1 ng/m^3^ (Fig. 4H). Excluding outlying temperature, the concentrations of TVOCs slightly increased as temperature increased from about 22 to 24.5 °C (Fig. 4D). When the temperature was greater than 23.5 °C, the concentrations of p-PAHs slightly increased as the temperature increased (Fig. 4I). The highest partial average level of TVOCs and p-PAHs was occurred with indoor activity “tobacco smoking” and the difference from other activities was 0.07 ppm and 6.5 ng/m^3^, respectively (Fig. 4E,J).

**Figure 5:**
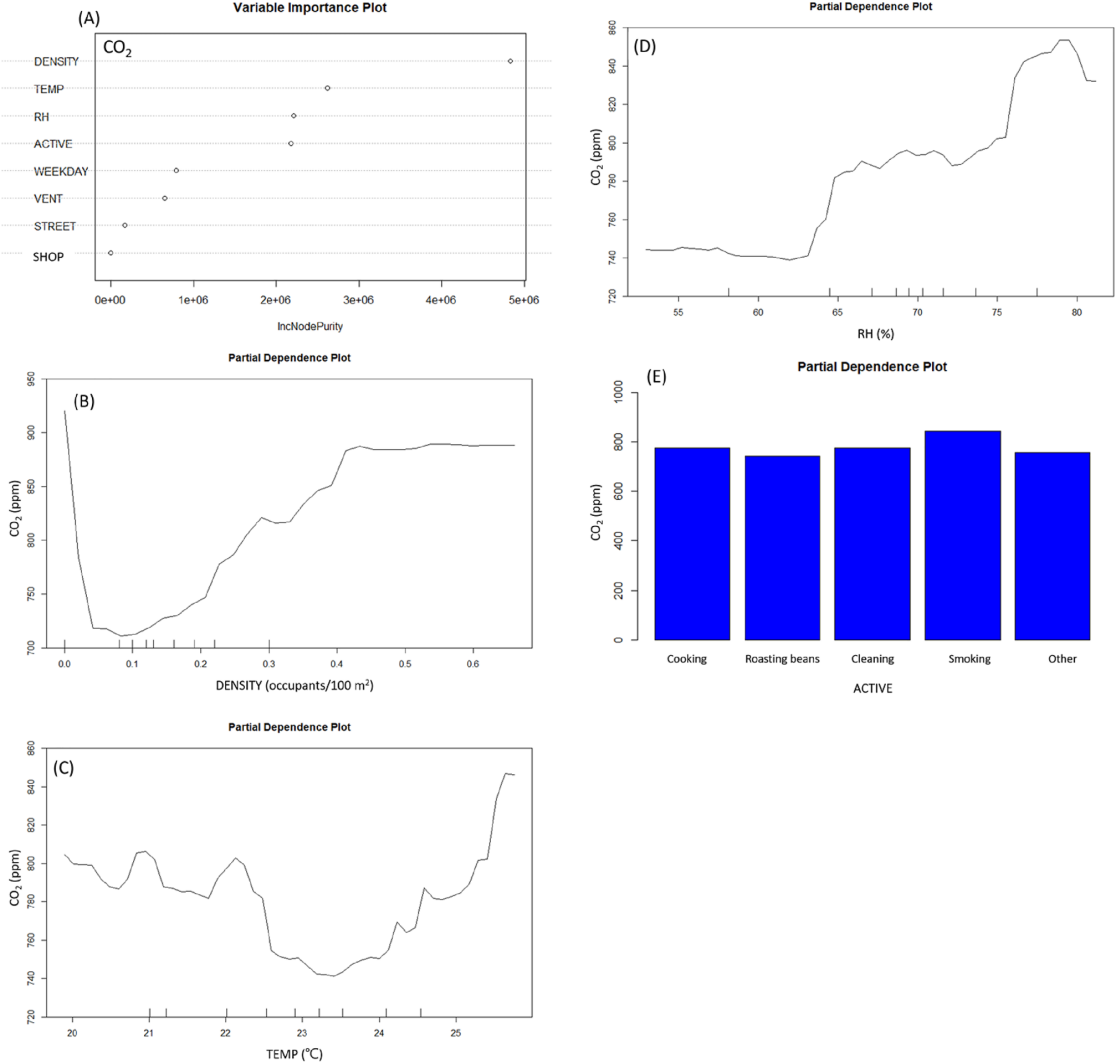
Variable importance rank and partial dependence plot of carbon dioxide (CO_2_) from Random Forests models. (ACTIVE, Indoor activities; CO_2_, carbon dioxide(ppm); DENSITY, Occupant density (person/m^2^); RH, relative humidity (%); SHOP, coffee shop A, B, C, D; STREET, locating on the main traffic street; TEMP, temperature(°C); VENT, ventilation status).

The top four indicators that affect the concentrations of CO_2_ were occupant density, temperature, RH, and indoor activity as shown in Fig. 5A and the R^2^ of RFs model was 0.53. Excluding outlying occupant density, the concentrations of CO_2_ significantly increased from about 723 to 885 ppm when the occupant density increased from about 0.08 to 0.43 person/m^2^ as showed in Fig. 5B. The relationship between temperature and CO_2_ was complicated and was basically shown in “U” shape. The bottom was at about 23.5 °C, when the temperature increased from 23.5 °C to about 25.8 °C, the concentrations of CO_2_ increased from about 740 to 848 ppm (Fig. 5C). Basically, CO_2_ was relatively low when RH was lower than 62%, but when the RH increased from about 62% to 80%, the concentrations of CO_2_ increased from about 740 ppm to 850 ppm (Fig. 5D). The highest partial average level of CO_2_ was occurred with indoor activity “tobacco smoking” and the difference from other activities was about 100 ppm (Fig. 5E).

## Discussion

### Compliance with regulations

In Taiwan, on average, each person drank 104 cups of coffee in 2020 according to annual coffee bean import statistics from the International coffee organization (ICO). Taiwan’s coffee shop density is the highest globally, and coffee chains Starbucks and Louisa have both exceeded 500 stores^51,52^. People might have more chances to stay at coffee shops in urban area. This environment is not only a living and leisure space, but also a workplace. However, the Taiwan IAQ regulation^47^ does not regulate these shops up till now. Customers won’t carry any IAQ instruments usually. The aim of this study was to identify the observable factors that can be the significant indicators of IAQ concurrently. The time of spending in a coffee shop is ranged from 4 to 6 h for working or studying to 15 min for grabbing a cup of coffee for to-go. So, we decided to inspect the 15-min average concentrations to reflect the exposure of group with short staying periods and also an assurance for all groups. These time-weight averages were applied to check the compliance with the IAQ standards/guidelines. Among all investigated café, 11.2%, 18.2%, and 21.1% of the 15-min averages of PM_10_, PM_2.5_, and CO_2_ accordingly did not meet the WHO guidelines^48^ and 8.3% of TVOCs exceeded Taiwan IAQ standard^47^. However, these comparisons could only be a reference, as IAQ standard regulated the time-weighted concentration of 1 h, 8 h or 24 h. Our results found the exceedances of short term (15-min) concentration remind the long-term time weighted average might underestimate the exposure of customers and employee at certain periods. The IAQ of café should be addressed as a workplace and/or a public environment to compliance with the regulations and to assure the healthy environments of people in these indoor spaces.

Cooking and bakery are the main sources of indoors’ PAHs. In coffee shop C and D, the p-PAHs were quantified in the range of 2.6 and 193.7 ng/m^3^ resulting from preparing the light meals. Levy et al*.*^5^ reported the p-PAH concentrations inside the coffee shop was 5–12 ng/m^3^. Abdullahi et al.^53^ reviewed the cooking emission studies and found the PAHs concentrations were variated with cooking styles, ranged from 0.2 to 1590 ng/m^3^. Ielpo et al. reported the mean concentration was 7.4 ng/m^3^ (range: 5–10 ng/m^3^) from bakery^54^. The differences may due to various characteristics of the PAH generating sources such as raw food types, cooking oil, cooking style and temperature^53,55^ and the indoor environment (dimensions and ventilation). No measurements of the gaseous PAHs also caused the underestimation.

### Significance of ranking

This study is the first one to rank the importance of indoor environmental factors and examine the relationships between factors and IAPs in independent coffee shops as novel workplace. Results of this study showed that occupant density, indoor activities, CO_2_ concentration, and temperature can remind the concentration changes of PM_10_, PM_2.5_, TVOCs, p-PAHs, and CO. Limited researches have evaluated the determinants of IAP in coffee shops. In this study, occupant density was found being the most important determinant of the indoor concentrations of PM_10_, PM_2.5_, TVOCs, p-PAHs, and CO_2_. On the other hand, the occupant density was the third and fourth important determinant of the concentrations of CO and PM_2.5–10_. We found slightly positive dose–response relationship between occupant density and air pollutants, particularly in the increasing of CO_2_ concentration. This was also found in other peer studies^26,56^. Previous study showed that the lower occupant density, the lower the PM concentration in preschools classrooms^18^, but this study didn't find dose–response effects between occupant density and concentrations of PM_10_, PM_2.5_, or PM_2.5–10_. The higher occupant density, the higher the concentrations of specific VOC^17^. We found the occupant density was the most important indicator for TVOCs. Coffee shops serve different functions for metropolitan people, such as social gatherings, studying, working and business meetings. Most of the shop design was the open-kitchen style, no significant segregation between dining and cooking areas. According the ANSI/ASHRAE standard 62.1, these investigated independent cafés were fitted in the occupancy category, cafeteria/fast-food dining and kitchen (cooking) both. While, the default occupant density with recommended ventilations of these two categories are different^49^. So more empirical researches are recommended to assure the appropriate occupant density of these shops to compliance with ASHRAE recommended air class.

Indoor activities were the second important determinant of the concentrations of PM_10_, PM_2.5_, and CO. For TVOCs, p-PAHs, and CO_2_, the indoor activities were the fourth important determinant. Besides, this study further pointed out the major human activity associated IAQ was tobacco smoking. Previous studies showed that concentrations of IAPs, such as VOCs^1,11^ PM_2.5_^1,13^, p-PAHs^1^, and CO^57^, were associated with tobacco use. Only shop C allowed indoor smoking among the 4 investigated shops. The partial dependence effect of smoking in increasing the pollutant concentrations were stronger than other indoor activities by RFs modeling with other environmental factors being controlled. Thus, it is important to ban the indoor smoking to reduce IAPs in coffee shops. Moreover, previous study pointed out the relationship between roasting coffee beans and concentration of CO^9^, this study further showed that roasting beans was a more important human activity associated with indoor CO concentration than tobacco smoking. As due to a limited number of studied coffee shops, this result need further confirmed.

CO_2_ is a global indicator of IAQ and a rough indicator of the effectiveness of ventilation. High CO_2_ level implies the possibilities of indoor IAPs accumulations^23,26–28^, our results showed that the concentrations of PM, TVOCs, p-PAHs, CO were proportional to the concentration of CO_2_. This confirmed the findings of other studies^21,23,42^. Besides, we found that the CO_2_ were the top three important predictors of the concentrations of multi-size PM, TVOCs, p-PAHs, and CO. The partial dependence plots indicated that the concentrations of IAPs increased proportional to the CO_2_ concentration once it exceeded 1000 ppm (Taiwan IAQ standard of 8-h average CO_2_). So, we recommend the low-cost CO_2_ monitor shall be installed in coffee shops to monitor CO_2_ and alert the IAQ. CO_2_ was affected by the occupants. However, RFs modeling can overcome the collinearity of CO_2_ and occupant to ranking the importance of these two determinants.

Temperature and RH are the important factors of thermal comfort. Previous studies reported that temperature and RH were related to the IAPs levels positively^19–21,23,39–42^. We found that the temperature was the top four important predictors of the concentrations of multi-size PM, TVOCs, p-PAHs, CO, and CO_2_. Relationships between indoor pollutants concentrations and temperature were complicated^19^, and the partial dependence plots of RFs models in our study show that the IAPs concentrations were consistently increased as the temperature increased within a certain range. The air conditioners were turned on during the business hours of these investigated shops. So, the temperature was kept constant with small variation (range 19.9–25.8 °C). The results were complied with the IAQ standards/guidelines of major Asian countries. Therefore, if temperature was selected to be an indicator to alert the levels of IAQ, future research should include the indoor temperature with big variation and be caution of the non-linear relationship with the IAP concentrations.

RH is less important than temperature to be an indicator of IAQ, and our partial dependence plots results showed the complicated non-linear relationships between IAPs and RH. We observed the RH was the most important predictor of the PM_2.5–10_ concentrations. PM_2.5–10_ decreased slightly as the RH increasing. Oliveira et al. found the concentrations of CO_2_, PM_1_, PM_2.5_, and PM_10_ were affected by the RH inversely in the kindergartens^21^. Some studies reported the positive correlations between RH and IAPs (e.g.CO_2_, HCHO, and TVOCs)^21,23,26^. The relationships were inconsistent among different researches. As we found, the relationship between RH and IAPs was complicated and nonlinear. RH is not an appropriate indicator for IAPs. Still, RH is relevant on the IAQ study, because it affects perceived IAQ comfort, synergistic effects may occur with air pollutants as well^58^. High RH provides the optimal condition for bacteria, fungi and viruses proliferation^59^. Indoor RH is not easy to control in Taiwan’s subtropical climate. Our monitoring data of coffee shops resulted in 42.6% of the RH over 70%. Currently, the IAQ standards or guidelines of Asian and European countries for temperature and RH criteria are different^49^. The RH of coffee shops shall be maintained within a comfort range according to the climate conditions.

The factors, weekday, ventilation status, shop’s pattern, and locating on the main street were less important in predicting the IAPs as they were not listed by the rfcv module in RFs model analysis. However, it did not mean that they had no effects of IAQ. The possible reasons were due to the small variations of the four investigated shops. For example, only three situations of the ventilation status were observed (Table 2), so the significance of these factors can’t be identified by the statistics analysis.

### Limitations and strength

Four unique café were investigated in this study. None of them were identical. The challenge of small sample size was compensated by RF models to illustrate the complicate non-linear relationship between IAPs and the determinant variables with limited numbers of data.

Meanwhile, the RFs model calculation considered all environmental factors simultaneously and provided insight in potential causal relationship between IAPs and environmental factors, particularly the temperature and RH. The total picture and interrelationships between different enironmental parameters were illustrated. On the other hand, our RFs model identified the most important determinat of CO_2_ concentraiotn was occupant density and the most important indoor activity in affecting PM_10_ and PM_2.5_ levels was occurred with “smoking”. A Rome’s study reported the tobacco smoking increased the indoor PM_2.5_ concentrations by two to three times of the non-smoking sites^1^. This was consistent with the present study and proved the reliablility of the RFs analysis results. More café should be included to validate the RFs models in the future.

### Recommendations

Although the strength of this study is that it used RFs models to examine indoor environmental indicators for reminding levels of IAQ in novel independent coffee shops, there still were some limitations. First, some studies showed that ventilation could influence IAQ, but this study didn’t measure outdoor air change rate in these participated shops. However, previous studies showed indoor ventilation was non-significantly associated with concentration of CO and PM^60,61^. Moreover, previous findings showed natural ventilation, such as window opening, and outdoor air pollutants would influence IAQ^19,60^. Therefore, it is suggested that future researchers could consider outdoor air pollution when investigating IAQ if there are natural ventilation in coffee shops. In addition, this study did not include chain coffee shops. In Taiwan, most of the chain coffee shops are located at the commercial buildings with central air conditioning systems which are different from the independent café with individual air-conditioners of this study. More coffee shops with different air-conditioning designs will be included in our future studies to validate and extend the applicability of the results of this study. Second, only four independent coffee shops participated in this study. Hence the findings cannot be inferred to other types of coffee shops. The importance ranking of determinants that affect IAPs may be changed due to the large variations of environmental factors if various types of coffee shops are included in future study. Last, our sampling time did not include the summer season, future study should evaluate potential seasonal variations and their influence.

## Conclusions

The application of RFs models in assessing and ranking the environmental factors that affect the IAPs of independent coffee shops was demonstrated. Meanwhile, the RFs was able to illustrate the complicated non-linear relationship between IAPs and determinant variables. Customers and staffs in the independent coffee shops can be reminded the change of indoor concentrations of PM, CO, CO_2_, TVOCs, and p-PAHs by observing the occupant density and human activities, such as tobacco smoking and roasting beans. Monitoring CO_2_ and maintaining the room temperature at appropriate range could also be the surrogate parameters to assure the acceptable IAQ.

## Supplementary Information

Supplementary Information 1.

Supplementary Information 2.

Supplementary Information 3.

## Data Availability

All data generated or analyzed during this study are included in this published article and its supplementary information files (Raw data coding.cvs and Raw data set.cvs).
