# Supplementary material for: Ranking the environmental factors of indoor air quality of metropolitan independent coffee shops by Random Forests model
Source: Sci Rep. 2022 Sep 26;12:16057. doi: 10.1038/s41598-022-20421-2 (PMC9513105; doi:10.1038/s41598-022-20421-2)
Supplement: Supplementary file 1 — Supplementary Information 1. [file 41598_2022_20421_MOESM1_ESM.docx]

Supplement A General descriptions of the coffee shops.

| **Shop** | **Building characteristics** | **Ventilation facilities** |
| --- | --- | --- |
| A | Indoor area: 806 m^3^  Opening hour: 7:00-22:00  locating on the main traffic street: yes  ETS-free area: yes  Kind of building: apartment  Floor: 1st floor  Types of the services: light meals and coffee  Interior Design Materials & Finishes: laminate | Air condition: 7 independent air conditioners  Ventilation: no window, one range hood for kitchens, exchange of outdoor air by door opened  Dominated natural ventilation (DNV) |
| B | Indoor area: 95 m^3^  Opening hour: 10:00-18:30  locating on the main traffic street: no  ETS-free area: yes  Kind of building: single house  Floor: 1st floor  Types of the services: coffee  Interior Design Materials & Finishes: conerete | Air condition: one independent air conditioners, one fan  Ventilation: 3 windows, keep windows and door opened during business hours  Dominated natural ventilation (DNV) |
| C | Indoor area: 182 m^3^  Opening hour:  (weekday) 11:00-20:00  (weekend) 11:00-01:00  locating on the main traffic street: no  ETS-free area: yes (daytime)  ETS area: yes (night time)  Kind of building: single house  Floor: 1st floor  Types of the services: light meals and coffee  Interior Design Materials & Finishes: conerete and ceramic tile | Air condition: 2 independent air conditioners, one fan, one air purifier  Ventilation: no window, one range hood for kitchens, exchange of outdoor air by door opened  Dominated natural ventilation (DNV) |
| D | Indoor area: 100 m^3^  Opening hour: 10:00-18:30  locating on the main traffic street: no  Smoking indoors: banned  Kind of building: single house  Floor: 1st floor  Types of the services: light meals and coffee  Interior Design Materials & Finishes: conerete and ceramic tile | Air condition: 2 independent air conditioners, one fan, one air purifier  Ventilation: no window, one range hood for kitchens, keep door opened during business hours  Dominated natural ventilation (DNV) |

ETS, environmental tobacco smoke.
